# Supplementary material for: The causal relationship between the human gut microbiota and pyogenic arthritis: a Mendelian randomization study
Source: Front Cell Infect Microbiol. 2024 Nov 26;14:1452480. doi: 10.3389/fcimb.2024.1452480 (PMC11629706; doi:10.3389/fcimb.2024.1452480)
Supplement: Supplementary file 1 [file DataSheet1.zip › mendelian test/ebi-a-GCST90027509.csv_forest.pdf]

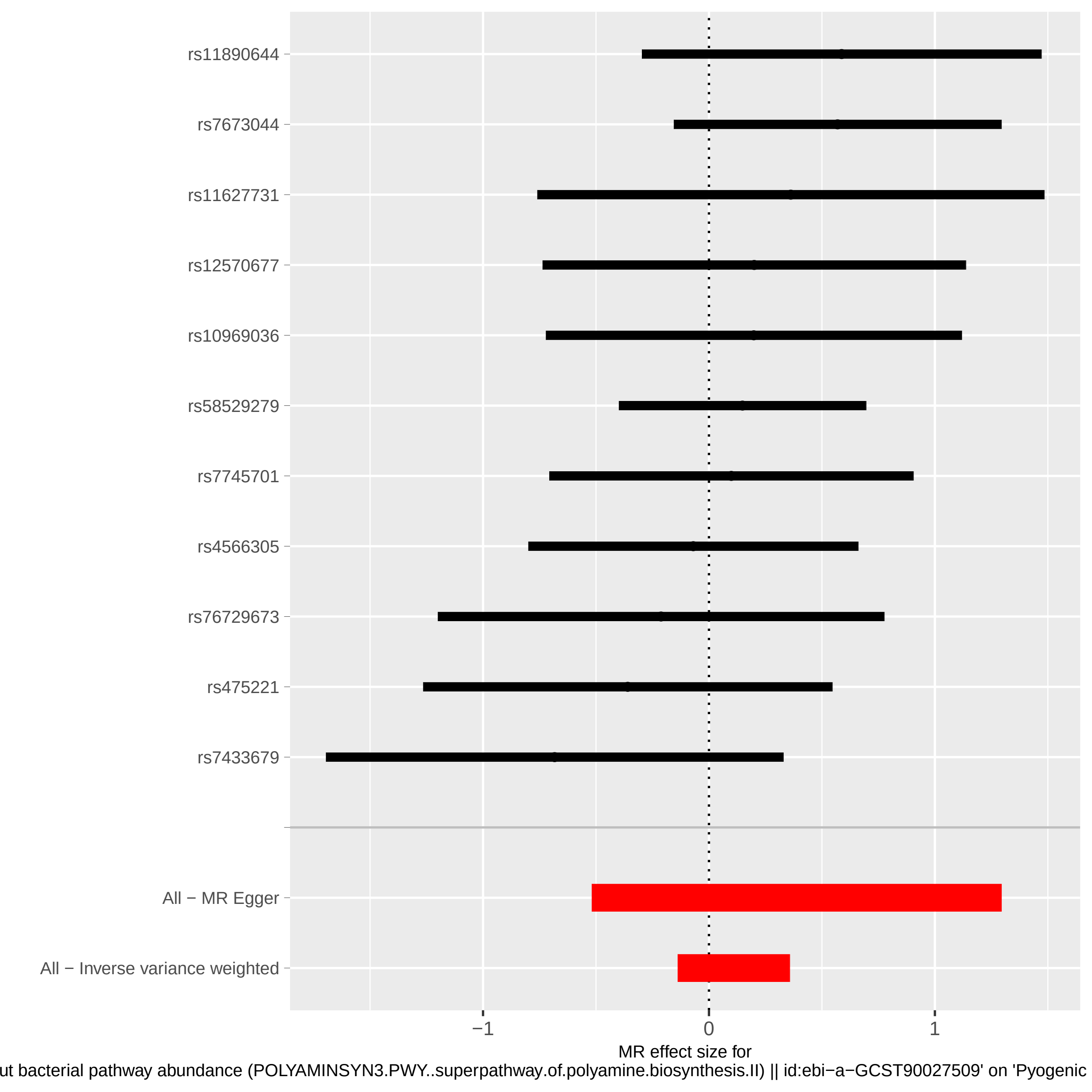
rs11890644
rs7673044
rs11627731
rs12570677
rs10969036
rs58529279
rs7745701
rs4566305
rs76729673
rs475221
rs7433679
All − MR Egger
All − Inverse variance weighted
−1
0
1
MR effect size for
ut bacterial pathway abundance (POLYAMINSYN3.PWY..superpathway.of.polyamine.biosynthesis.II) || id:ebi−a−GCST90027509' on 'Pyogenic
